# Hepatitis C Virus Stimulates Murine CD8α-Like Dendritic Cells to Produce Type I Interferon in a TRIF-Dependent Manner

**DOI:** 10.1371/journal.ppat.1005736

**Published:** 2016-07-06

**Authors:** Stephanie Pfaender, Elena Grabski, Claudia N. Detje, Nina Riebesehl, Stefan Lienenklaus, Eike Steinmann, Ulrich Kalinke, Thomas Pietschmann

**Affiliations:** 1 Institute for Experimental Virology, TWINCORE Centre for Experimental and Clinical Infection Research; a joint venture between the Medical School Hannover and the Helmholtz Centre for Infection Research, Hannover, Germany; 2 Institute for Experimental Infection Research, TWINCORE, Centre for Experimental and Clinical Infection Research, a joint venture between the Hannover Medical School and the Helmholtz Centre for Infection Research, Hannover, Germany; 3 Molecular Immunology, Helmholtz Centre for Infection Research, Braunschweig, Germany; 4 Institute for Laboratory Animal Science, Hannover Medical School, Hannover, Germany; Purdue University, UNITED STATES

## Abstract

Hepatitis C virus (HCV) induces interferon (IFN) stimulated genes in the liver despite of distinct innate immune evasion mechanisms, suggesting that beyond HCV infected cells other cell types contribute to innate immune activation. Upon coculture with HCV replicating cells, human CD141^+^ myeloid dendritic cells (DC) produce type III IFN, whereas plasmacytoid dendritic cells (pDC) mount type I IFN responses. Due to limitations in the genetic manipulation of primary human DCs, we explored HCV mediated stimulation of murine DC subsets. Coculture of HCV RNA transfected human or murine hepatoma cells with murine bone marrow-derived DC cultures revealed that only Flt3-L DC cultures, but not GM-CSF DC cultures responded with IFN production. Cells transfected with full length or subgenomic viral RNA stimulated IFN release indicating that infectious virus particle formation is not essential in this process. Use of differentiated DC from mice with genetic lesions in innate immune signalling showed that IFN secretion by HCV-stimulated murine DC was independent of MyD88 and CARDIF, but dependent on TRIF and IFNAR signalling. Separating Flt3-L DC cultures into pDC and conventional CD11b-like and CD8α-like DC revealed that the CD8α-like DC, homologous to the human CD141^+^ DC, release interferon upon stimulation by HCV replicating cells. In contrast, the other cell types and in particular the pDC did not. Injection of human HCV subgenomic replicon cells into IFN-β reporter mice confirmed the interferon induction upon HCV replication *in vivo*. These results indicate that HCV-replicating cells stimulate IFN secretion from murine CD8α-like DC independent of infectious virus production. Thus, this work defines basic principles of viral recognition by murine DC populations. Moreover, this model should be useful to explore the interaction between dendritic cells during HCV replication and to define how viral signatures are delivered to and recognized by immune cells to trigger IFN release.

## Author Summary

HCV is an RNA virus that, following exposure, in most cases establishes chronic infection. The virus has evolved numerous immune evasion strategies, including direct interference with interferon production. Nevertheless, HCV infection activates interferon-stimulated genes in the liver, implying that non-infected cells secrete IFN. Several DC subsets have been implicated in HCV sensing and production of IFN; however, the molecular mechanism resulting in HCV sensing is poorly understood. Using murine bone marrow derived DC, we dissected basic principles of HCV innate immune recognition and activation of dendritic cells. We show that HCV recognition by murine DCs depends on TRIF and IFN receptor signalling. This indicated the involvement of TLR3 and of the IFN receptor dependent amplification loop. Infectious virus production is dispensable since cells carrying subgenomic HCV replicons are also recognized. Moreover, specific DC subtypes, i.e. CD8α-like DC, are responsible for recognition of HCV. These findings highlight that specific murine DC subpopulations are uniquely capable of recognizing HCV replicating cells independent of infectious virus production. These observations open novel opportunities to explore the mechanisms of inter-cellular communication that mediate activation and IFN production of non-infected immune cells and to dissect the role of DC subsets in immune control.

## Introduction

Hepatitis C virus (HCV) infection constitutes a major global health problem since more than 140 million people suffer from chronic sequelae of the infection [1]. Once infected, approximately 80% of the individuals are not able to clear the pathogen and develop a chronic infection that often is associated with liver diseases such as fibrosis, cirrhosis and hepatocellular carcinoma, thus resulting in the need of liver transplantation [2]. It is believed that chronic infections are a consequence of a multi-factorial immune failure, due to delayed and weak T-cell responses, as well as dysfunctional B-cell, natural killer (NK) -cell and dendritic cell (DC) responses [3–9]. In addition, it has been shown that a strong pre-stimulation of interferon (IFN) stimulated genes (ISGs) during chronic HCV infection constitutes a marker of decreased responsiveness to IFN-based therapies [10]. Bridging the innate with the adaptive immune responses, DC have an important role in the establishment of a protective immune response and they are crucial for the production of interferons and the activation of immune cells [11]. Based on their distinct phenotype and functional characteristics, human peripheral DC can be classified in 3 major subsets. These include conventional DC (cDC) which encompass the myeloid CD1c^+^/BDCA1^+^ DC (mDC1) that are the largest mDC population in the blood and that are known for their antigen presenting capacity and cytokine expression, and the myeloid CD141^+^/BDCA3^+^ DC (mDC2) which produce IL-12 and type III IFN and have the ability to cross-present antigens to CD8 T-cells. The third subset is represented by the plasmacytoid dendritic cells (pDC), also known as natural type I IFN-producing cells, which upon activation produce high levels of type I IFN [12–15]. All three subsets have been implicated to be involved in responses to HCV infection [16–20]. However, not much is known about the interplay between the DC subsets among each other and with other immune cells. This is at least in part due to technical as well as practical difficulties of studying human DC. Studies of murine DC principally offer an attractive alternative for dissecting the requirements of DC stimulation. However, due to the restricted species tropism of HCV to humans and the lack of suitable immune competent mouse models [21] not much research has been performed using murine dendritic cells. A clear advantage of the murine system is the availability of genetically modified animals that allow in depth mechanistic studies. Furthermore, homologs of human dendritic cell subsets can be easily generated upon addition of FMS-like tyrosine 3 ligand (Flt3-L) to murine bone marrow (BM) cells, thus inducing high numbers of BM-derived DC [22–25]. Likewise, murine DC can be generated from BM using granulocyte/macrophage colony-stimulating factor (GM-CSF), whereas these cell cultures lack the pDC counterparts and are believed to yield DC which primarily resemble monocyte-derived DC [24, 26, 27]. To some extent Flt3-L DC cultures reflect the physiological DC development that gives rise to three major DC subsets for which orthologues can similarly also be found in the human peripheral blood: pDC as well as the two subsets of conventional dendritic cells, CD8α-like DC which resemble the human CD141^+^ (mDC2) subset and CD11b-like DC which are homologous to the human CD1c^+^ DC (mDC1) [23, 28]. Given the homology between murine and human DC subsets we used murine Flt3-L differentiated DC from wildtype as well as genetically modified mice to dissect the requirements for DC stimulation by HCV.

## Results

### Flt3-L but not GM-CSF differentiated dendritic cells mount type I and type III IFN responses after coculture with HCV transfected cells

**Fig 1:**
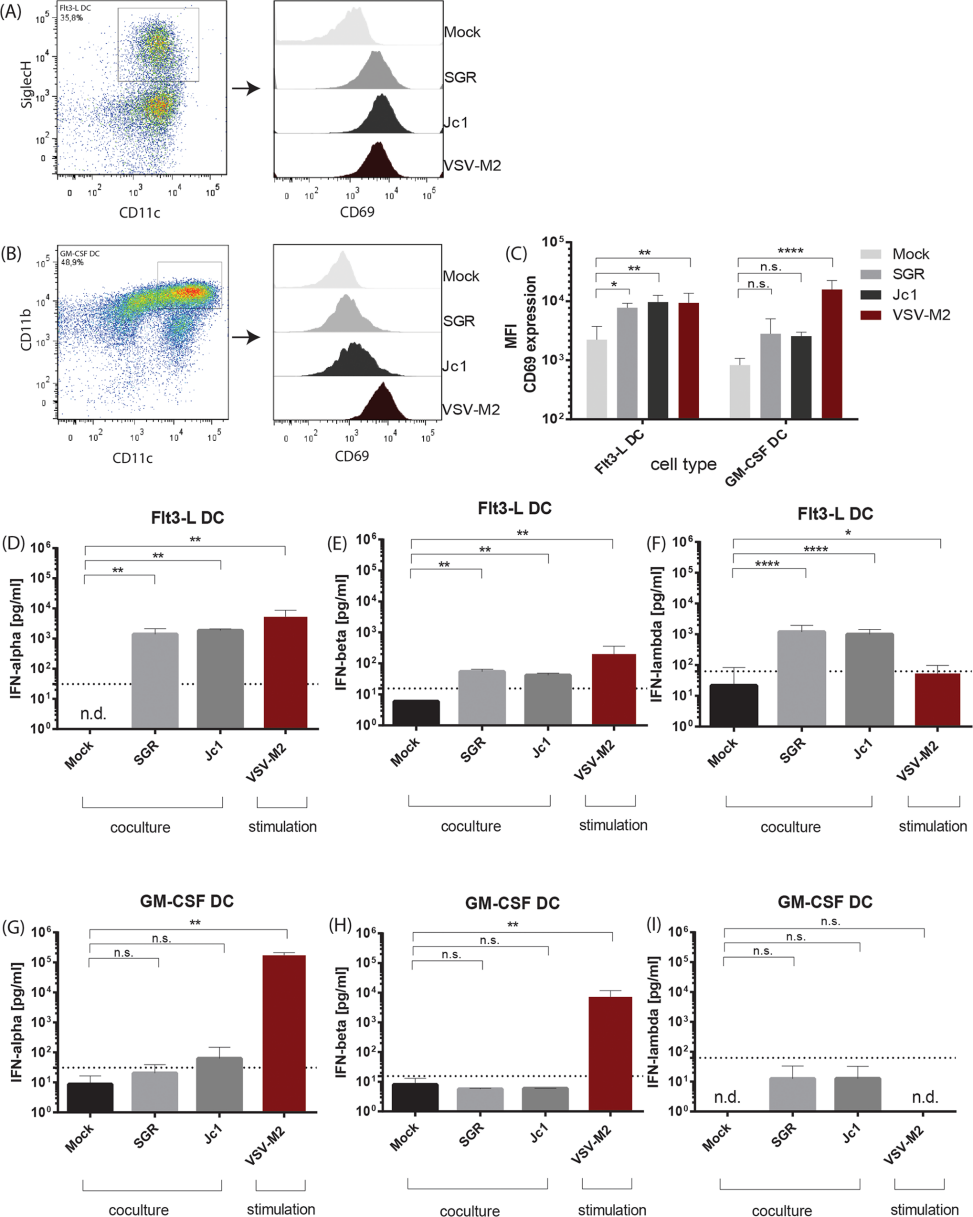
Flt3-L derived DC, but not GM-CSF mDC, are activated after coculture with HCV transfected hepatoma cells and produce type I and type III IFN. Huh7.5 cells were transfected with HCV subgenomic replicon (SGR) RNA or HCV full length (Jc1) RNA and incubated for 72 h. Murine Flt3-L derived DC or GM-CSF derived DC were cocultured with mock or HCV RNA transfected hepatoma cells or stimulated with VSV-M2 at a MOI 1 for 18 h and cells were analyzed by flow cytometry or ELISA. (A) Exemplary gating on SiglecH^+^ CD11c^+^ Flt3-L DC and analysis of the CD69 expression. (B) Exemplary gating on CD11c^+^ CD11b^+^ GM-CSF DC and analysis of the CD69 expression. (C) Quantification of the mean fluorescent intensity (MFI) of CD69 expression by Flt3-L DC or GM-CSF DC after coculture and stimulation (n = 3–4). Analysis of IFN-α (D), IFN-β (E) and IFN-λ (F) in cell-free supernatants of Flt3-L derived DC cultures by ELISA. Analysis of IFN-α (G), IFN-β (H) and IFN-λ (I) in cell-free supernatants of GM-CSF derived DC cultures (n = 3–6). Dashed line indicates the lowest value of the standard of the respective ELISA assay, n.d. not detected. (****, p≤ 0.0001, ***, p≤ 0.001; **, P≤0.01; *, P≤0.05; 2-way ANOVA, means + SD; n.s. not significant).

Murine bone marrow (BM) cells were isolated and *in vitro* differentiated into dendritic cells using medium enriched either with the cytokines Flt3-L or GM-CSF. Subsequently, cells were cocultured with HCV subgenomic replicon (SGR) or HCV full-length (Jc1) transfected human Huh7.5 cells for 18 hours (as further described in the materials and methods section) and analyzed by flow cytometry. In parallel, DC populations were stimulated with VSV-M2 at a multiplicity of infection (MOI) of 1. To assess the activation of the respective DC populations SiglecH^+^ CD11c^+^ Flt3-L DC and CD11c^+^ CD11b^+^ GM-CSF DC were analyzed for the expression of the activation marker CD69 using flow cytometry (Fig 1A and 1B). Cocultivation of HCV replicon or virus-transfected hepatoma cells with DC cultures led to an upregulation of CD69 expression on both cell types analyzed. This is evidenced by the representative FACS histograms depicted in Fig 1B and by the mean values of CD69 surface expression (MFI) across at least three independent experiments given in Fig 1C. Collectively, these results indicated that HCV replication was sensed by murine DC subsets and led to their activation.

Since activation occurred also upon co-culture with replicon-transfected cells, which do not produce infectious viral progeny, DC activation seems to be independent of virus assembly and release. To analyze the cytokine production, supernatants of DC cocultures were harvested and analyzed for the production of type I as well as type III IFNs by using commercially available ELISAs. Only Flt3-L derived DC cultures produced significant amounts of IFN-α (Fig 1D), IFN-β (Fig 1E) and IFN-λ (Fig 1F) in response to HCV replication, whereas GM-CSF-derived DC did not (Fig 1G–1I). Stimulation with VSV-M2 as control led to a significant type I IFN production from both cell types (Fig 1D, 1E, 1G and 1H) indicating that only Flt3-L derived DC are activated by HCV replication to produce significant amounts of type I and type III IFN.

### Type I IFN production by Flt3-L derived DC is RNA replication but not cell-cell contact dependent

**Fig 2:**
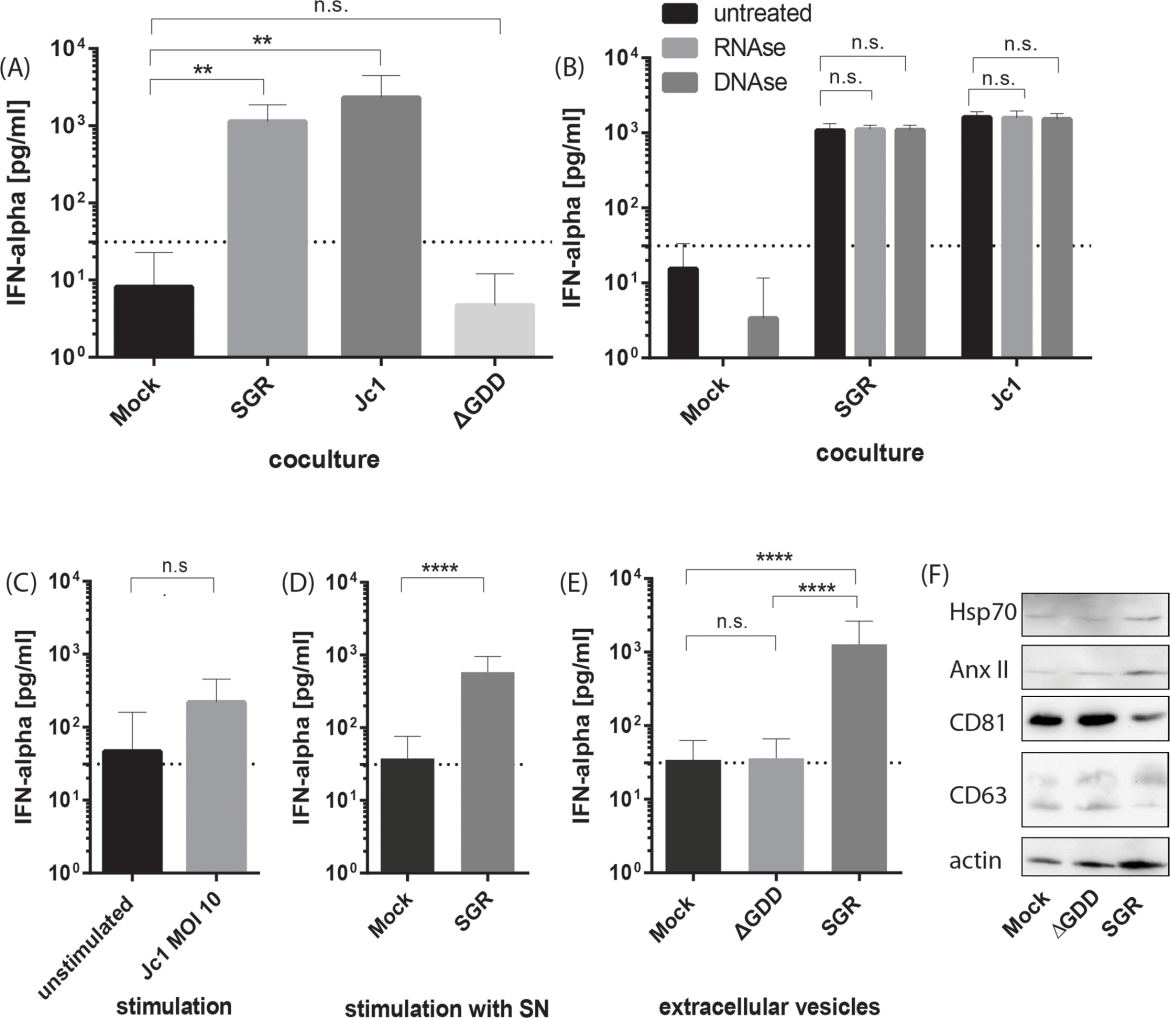
Type I IFN production by Flt3-L DC cultures is dependent on HCV RNA replication and independent of cell-to-cell contact. (A) Huh7.5 cells were mock transfected or transfected with SGR, Jc1 or Jc1ΔGDD (ΔGDD) RNA, co-cultivated with Flt3-L derived DC cultures and the amount of IFN-α in the supernatant was determined (n = 3). (B) Mock or HCV RNA transfected hepatoma cells were treated with 0.5 μg/mL RNAse or 1 unit DNAse before Flt3-L DC were added in a coculture. After 18 h, IFN-α was detected in the cell-free supernatants (n = 3). Flt3-L derived DC were seeded and stimulated with Jc1 (C) or 5 μL concentrated SN from Mock or HCV SGR transfected cells (D) (n = 3). (E) Extracellular vesicles were isolated from concentrated SN from Mock, pUCΔGDD (ΔGDD) or HCV SGR transfected cells. 5 μL of isolated vesicles were used to stimulate Flt3-L DC for 18 h and IFN-α was quantified in the cell-free supernatant by ELISA (n = 6). (F) Protein content of isolated extracellular vesicles was analyzed using antibodies against polypeptides typically enriched in exosomes (Hsp70, AnxII, CD81, CD63 and actin). Dashed line indicates the lowest value of the standard of the respective ELISA assay, n.d. not detected. (****, p≤ 0.0001, ***, p≤ 0.001; **, P≤0.01; *, P≤0.05; Mann-Whitney test and 2-way ANOVA, means + SD; n.s. not significant).

To further characterize the requirements for IFN release by HCV-stimulated Flt3-L derived DC, cells were cocultured with HCV cells transfected with the HCV subgenomic replicon (SGR), the full length virus (Jc1), or a replication incompetent mutant full length virus which encodes an in-frame deletion of 10 amino acids spanning the GDD motif that abolishes the RNA polymerase activity (ΔGDD). Both replication of the HCV SGR and the full length virus stimulated IFN-α production by Flt3-L derived DC, whereas coculture with transfected cells harboring the replication incompetent HCV construct did not result in detectable IFN production (Fig 2A). These experiments indicated that virus replication but not infectious virus production is necessary for the murine Flt3-L derived DC to sense HCV. Treatment with RNAse and DNAse during coculture confirmed that the activation of the DCs was not due to residual exogenous nucleic acids in the supernatant of the cultures (Fig 2B). To further define the mode of stimulation, either cell-free HCV virus (Jc1) containing culture fluid or concentrated supernatant (SN) of SGR transfected cells was used to stimulate the DC. Cell-free HCV preparations (Jc1 MOI 10) were incubated with 2x10^5^ DC for 18 h and secretion of IFN was quantified. As control, DCs were left untreated. As is evidenced in Fig 2C treatment with cell-free Jc1 slightly upregulated IFN production by dendritic cells compared to unstimulated cells. However, stimulation of Flt3-L derived DC with the culture fluid derived of SGR-transfected cells that was concentrated led to a statistically significant IFN production by dendritic cells (Fig 2D), indicating the cell-free stimulation of Flt3-L derived DC by HCV signatures released from replicon cells. Extracellular vesicles, like exosomes, have been implicated in the stimulation of human pDC [29]. To analyze whether extracellular vesicles could mediate stimulation of murine DC, we isolated vesicles from mock, replication incompetent HCV subgenomic replicon mutant (pUCΔGDD) ΔGDD or SGR transfected cells employing a commercial exosome purification kit and used these preparations to stimulate murine DC (Fig 2E). Western blot analyses confirmed enrichment of extracellular vesicles that contain typical markers of exosomes like CD81, CD63, Hsp70 and AnxII [30] (Fig 2F). Only vesicles derived from HCV replicating cells were able to stimulate the murine DC to produce type I IFN. These data indicate that cell-cell contact is not necessary for murine Flt3-L derived DC stimulation and that extracellular vesicles could contribute to DC mediated type I IFN production. As human DC have been described to recognize HCV replication *in vitro* in an endosome-dependent manner [17, 31] the cocultures were treated with the endosomal acidification inhibitors bafilomycin A1 or concanamycin A at a dose of 25 nM or 5, respectively, throughout the duration of the coculture. These treatments with inhibitors of endosomal acidification resulted in an abolishment of the IFN-α production, indicating that endosomal acidification is required for the production of type I IFN by murine DC (S1 Fig). Taken together, type I IFN production by murine Flt3-L derived DC is dependent on RNA replication but independent of infectious virus production and of direct cell-to-cell contact.

### TRIF signaling and type I IFN receptor signaling mediate the type I IFN production after coculture with HCV transfected cells

**Fig 3:**
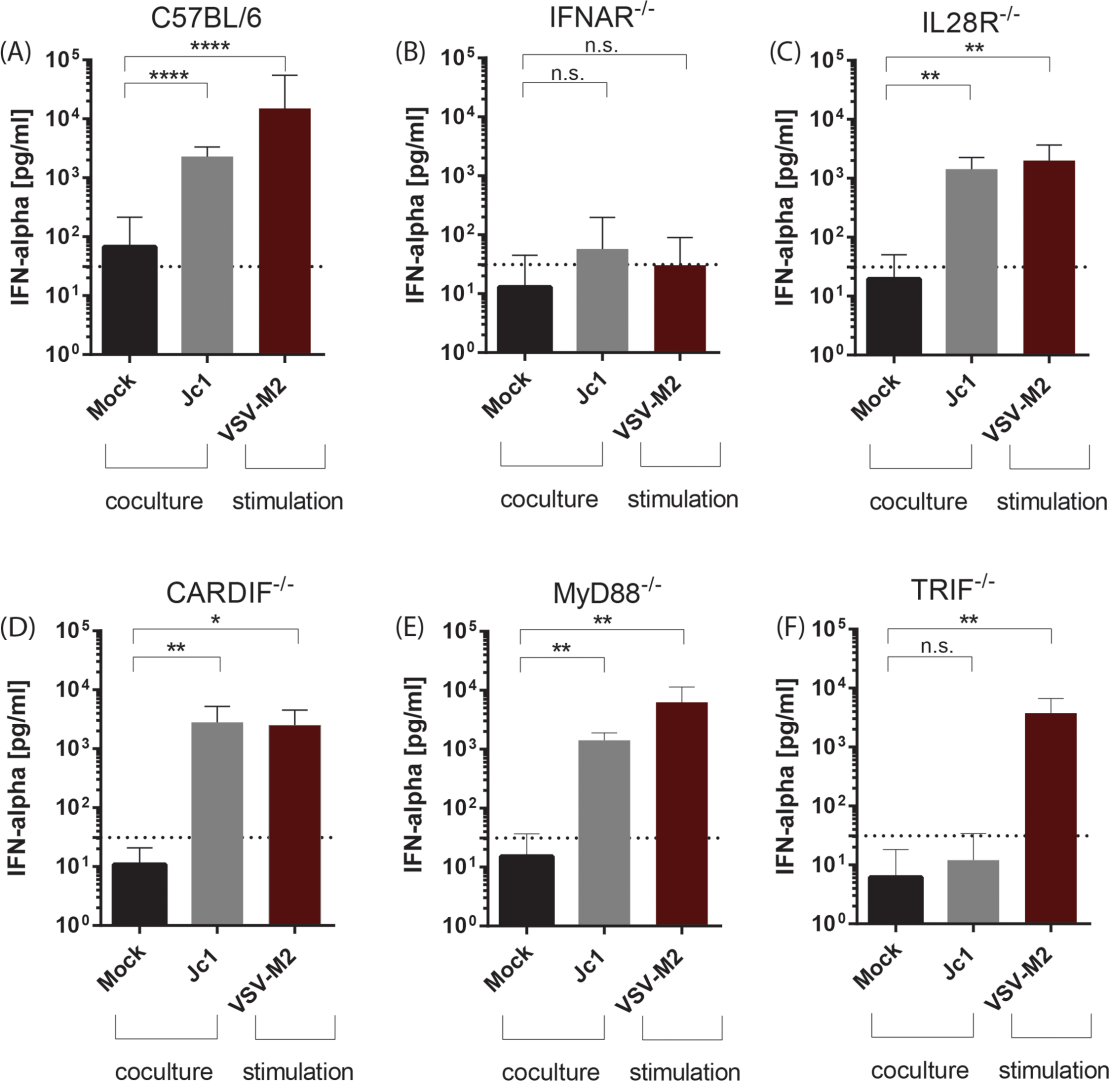
Type I IFN production is dependent on IFNAR and TRIF signaling. Flt3-L DC were generated from (A) C57BL/6, (B) IFNAR^-/-^, (C) IL28R^-/-^, (D) CARDIF^-/-^, (E) MyD88^-/-^ and (F) TRIF^-/-^ mice and cocultured with mock or HCV transfected cells or stimulated with VSV-M2 at a MOI 1. After 18 h, IFN-α was quantified in cell-free supernatant by ELISA (n = 3–8). Dashed line indicates the lowest value of the standard of the respective ELISA assay, n.d. not detected. (****, p≤ 0.0001, ***, p≤ 0.001; **, P≤0.01; *, P≤0.05; Mann-Whitney test, means + SD; n.s. not significant).

Human pDC have been described to sense HCV RNA via Toll-like receptor (TLR7) which signals via the adaptor molecule MyD88 [19, 32] whereas human CD141^+^ DC (mDC2) mainly use TLR3 and TRIF signaling [17, 18]. To define the immune sensing pathways of the murine DC counterparts, DCs from different knock out animals harboring genetic lesions in the type I IFN receptor (IFNAR), type III IFN receptor (IL28R) or the adaptor molecules CARDIF (also known as MAVS, IPS-1, VISA), MyD88 or TRIF were analyzed. As observed before, DC generated from wild type animals supported a significant amount of type I IFN production upon coculture with Jc1 transfected cells (Fig 3A). Genetic lesions in IL28R (Fig 3C), CARDIF (Fig 3D) and MyD88 (Fig 3E) had no influence on type I IFN production in these coculture assays. However, the lack of IFNAR (Fig 3B) or TRIF (Fig 3F) abrogated type I IFN production, indicating that the type I IFN feedback loop as well as TLR3-dependent signaling are important for the IFN production by Flt3-L derived DC after coculture with HCV transfected cells.

### CD8α-like dendritic cells sense HCV transfected cells and produce type I IFN

**Fig 4:**
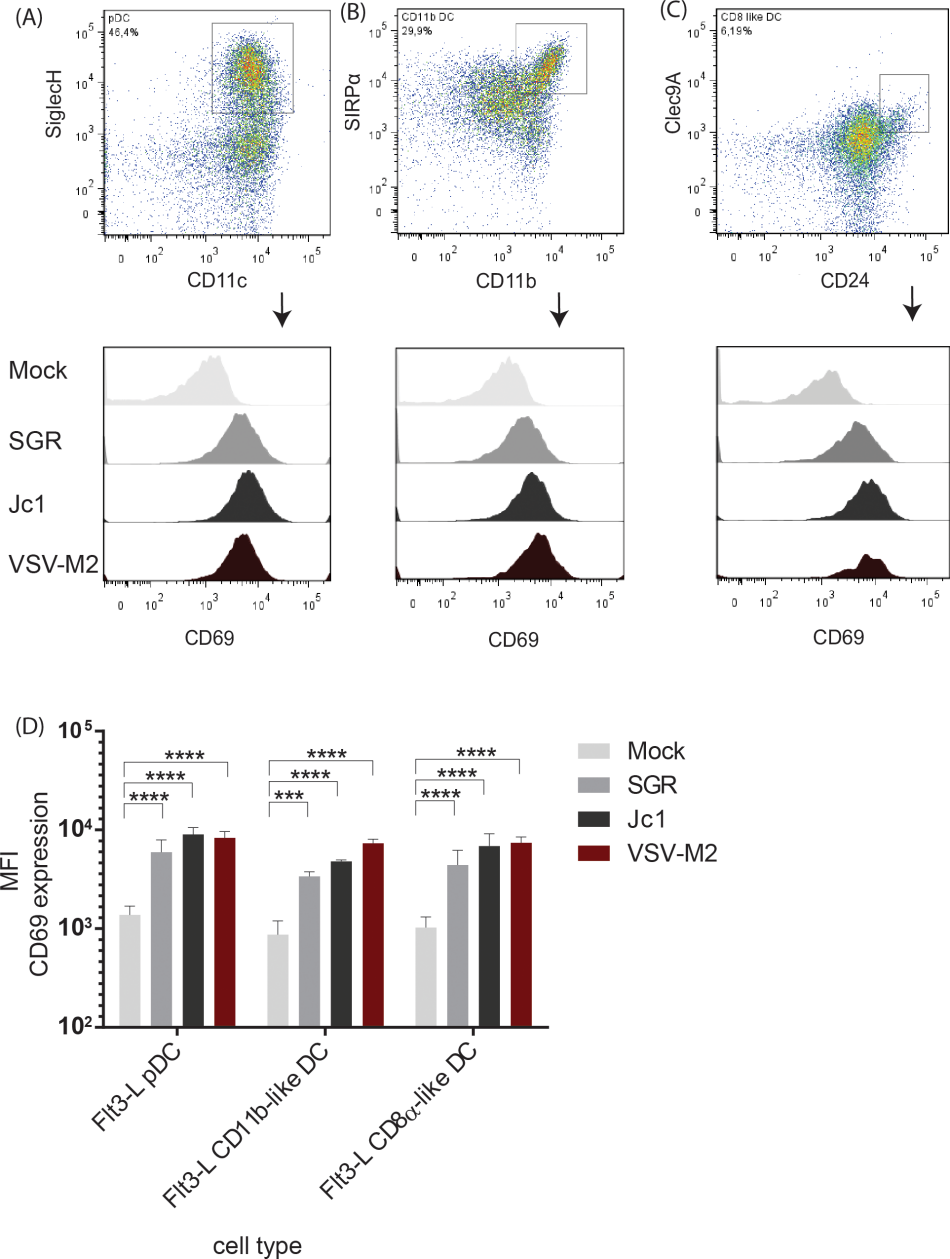
In the Flt3-L derived DC cultures pDC, CD11b-like DC and CD8α-like DC show enhanced CD69 expression after coculture with HCV transfected hepatoma cells. Flt3-L derived DC were co-cultivated with HCV transfected hepatoma cells or stimulated with VSV-M2 at a MOI 1 for 18 h and analyzed by flow cytometry. Cells were gated on (A) SiglecH^+^ CD11c^+^ pDC, (B) SIRPα^+^ CD11b^+^ CD11b-like DC or (C) Clec9A^+^ CD24^+^ CD8α-like DC and analyzed for the up-regulation of CD69 expression. (D) Quantification of the mean fluorescent intensity (MFI) of CD69 expression by Flt3-L derived pDC, CD11b-like DC and CD8α-like DC (n = 3) (****, p≤ 0.0001, ***, p≤ 0.001; **, P≤0.01; *, P≤0.05; 2-way ANOVA, means + SD; n.s. not significant).

To explore which cell subset within the Flt3-L DC culture is able to sense and respond to HCV replication additional surface marker were included for subsequent FACS analysis (Fig 4A–4C). Cells were stained with antibodies targeting SiglecH and CD11c to detect pDC, SIRPα and CD11b to detect CD11b-like DC or Clec9A and CD24 to analyze CD8α-like DC. Gating on the respective cell populations revealed that all three DC subsets, pDC, CD11b-like DC and CD8α-like DC, significantly upregulated the activation marker CD69 after coculture with HCV transfected cells (Fig 4D) indicating an involvement of all three DC subsets.

**Fig 5:**
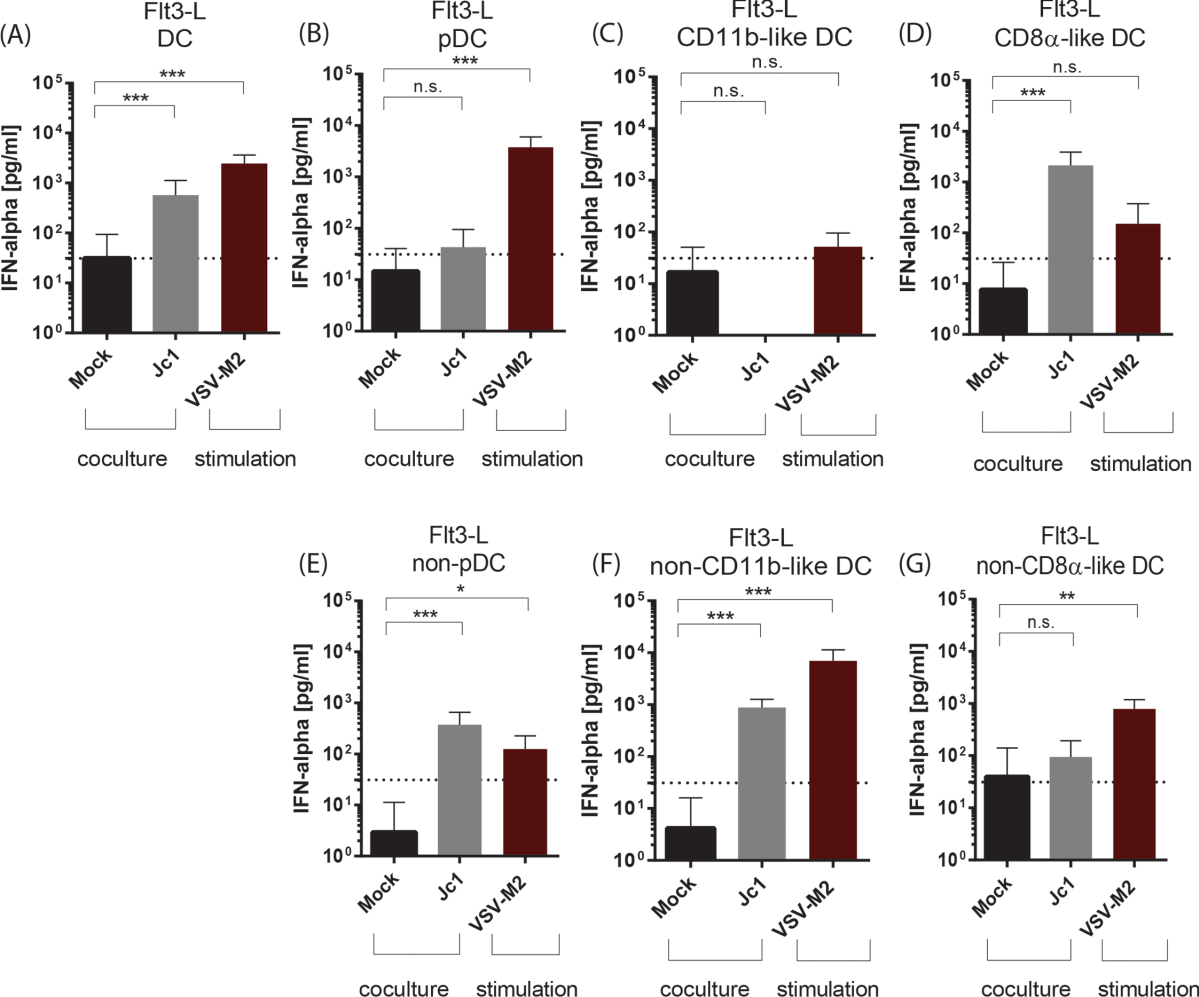
HCV replication stimulates Flt3-L CD8α-like DC to produce type I IFN. Flt3-L DC cultures were FACS sorted into pDC, CD11b-like DC and CD8α-like DC or the respective residual cells and co-cultured with mock or HCV transfected cells or stimulated with VSV-M2 at a MOI 1 for 18 h and the IFN-α amount in the supernatant measured by ELISA. Coculture with Flt3-L DC (A), sorted Flt3-L pDC (B), sorted CD11b-like DC (C), sorted CD8α-like DC (D), Flt3-L non-pDC (residual cells from pDC sort) (E), Flt3-L non-CD11b-like DC (residual cells from CD11b DC sort) (F) and Flt3-L non-CD8α-like DC (residual cells from CD8α-like DC sort) (G) (n = 4). Dashed line indicates the lowest value of the standard of the respective ELISA assay, n.d. not detected. (****, p≤ 0.0001, ***, p≤ 0.001; **, P≤0.01; *, P≤0.05; Mann-Whitney test, means + SD; n.s. not significant).

To further define the role of each subset, cells were FACS sorted into pDC, CD11b-like DC, and CD8α-like DC and independently cocultured with HCV transfected cells. As observed before, cocultivation of the complete Flt3-L derived DC population with HCV transfected cells led to a significant type I IFN production (Fig 5A). Separation of the DC subsets revealed that pDC (Fig 5B) and CD11b-like DC (Fig 5C) were not able to respond to HCV replicating cells. However, CD8α-like DC cocultured with HCV transfected cells produced significant amounts of type I IFN (Fig 5D). In line with this finding, depletion of pDC (non pDC, Fig 5E) or CD11b-like DC (non CD11b-like DC, Fig 5F) had no impact on the IFN production after coculture, whereas depletion of CD8α-like DC abrogated type I IFN production (non CD8α-like DC, Fig 5G) indicating that the CD8α-like DC are the main IFN producing cells within the Flt3-L culture after co-culture with HCV transfected cells. Consistent with previous observations, stimulation with infectious VSV-M2 triggered mainly the pDC to produce large amounts of IFN-α (Fig 5B), confirming the role of pDC as the main IFN producing cells after viral infection. Since human pDC have been described to induce a robust type I IFN production after coculture with HCV transfected cells, we analyzed whether the xeno-situation, namely adding murine DC to human liver cells, might be responsible for this lack of response. To this end, murine liver cells harboring a genetic lesions in MAVS (also known as CARDIF, IPS-1, VISA) and containing all minimal factors required for HCV replication and infection (MLT-MAVS^−/−^miR-122/mmmmm [33]), were transfected with HCV SGR RNA and cocultured either with the mixed Flt3-L culture or purified murine Flt3-L derived DC subsets. To increase viral replication in these cells, the HCV subgenomic replicon SGR2 (pFK;i341NeoEI-NS3-NS5B/JFH1) displaying a more robust replication, was utilized in addition. As seen before, the complete Flt3-L DC culture produced type I IFN after co-culture with either human or murine HCV transfected cells (S2A Fig and S2E Fig, respectively). Among the purified subpopulations, only CD8α-like DC were able to respond to HCV replication in human or murine cells (S2D Fig and S2H Fig, respectively), whereas all other cell types did not secrete elevated levels of IFN upon coculture with these HCV replicating cells (S2B and S2C Fig and S2F and S2G Fig, respectively). These experiments indicated that murine Flt3-L derived pDC are not able to sense HCV replication. In summary, murine CD8α-like DC, but not murine pDC or murine CD11b-like DC, sense HCV transfected cells and produce type I IFN.

### HCV transfected cells stimulate type I IFN induction *in vivo*

**Fig 6:**
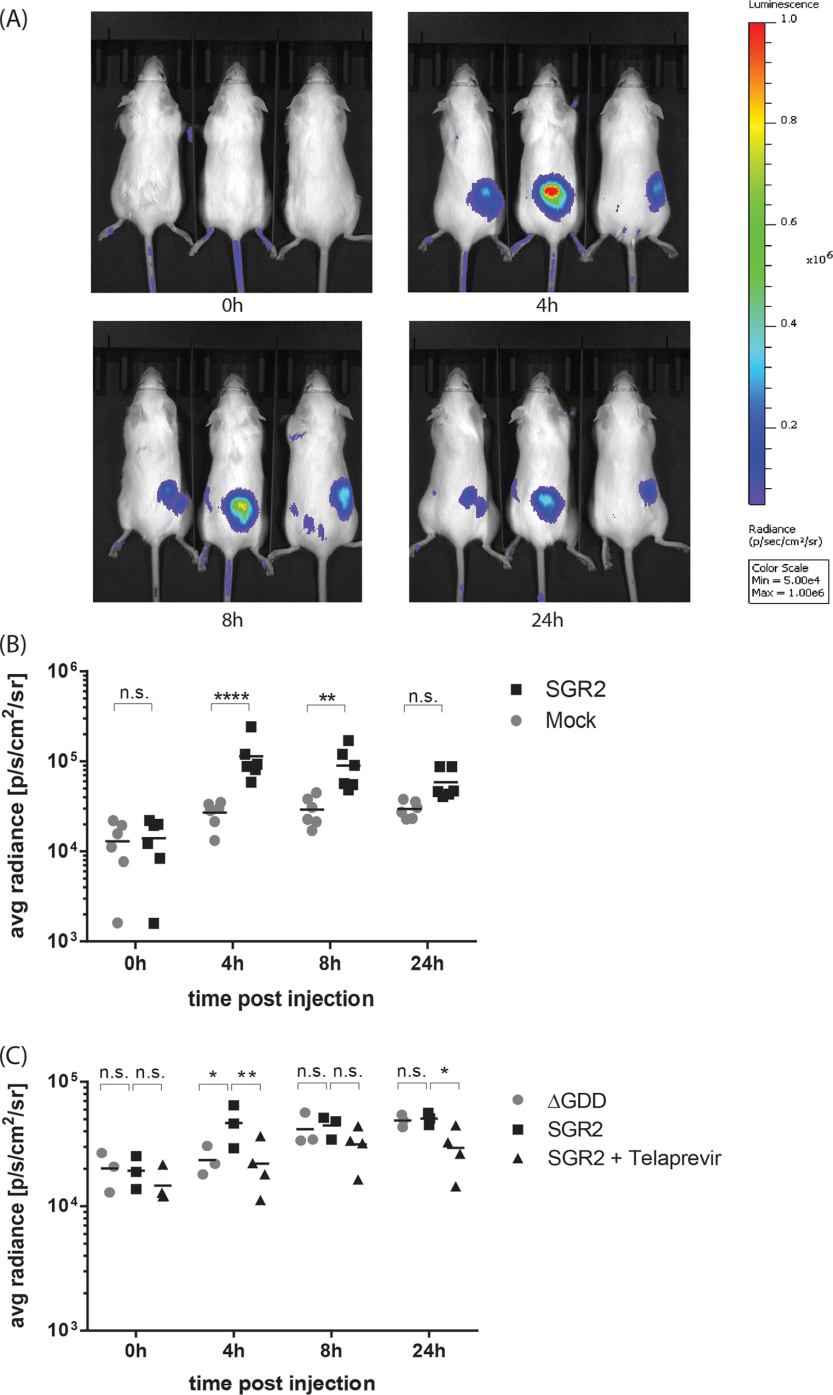
HCV-replicating cells induce IFN-β induction *in vivo*. (A) IFN-β^+/Δβluc^ mice were s.c. injected with 5x10^6^ mock transfected (left flank) or HCV subgenomic RNA transfected (SGR2; right flank) Huh7.5 cells. At the indicated time points post s.c. injection, luciferin was injected i.v. and luciferase activity was measured using the IVIS Spectrum *in vivo* imaging system. (B) Quantification of luciferase activity. Each symbol represents a region of interest (ROI) analysis of an individual animal (n = 6). (C) IFN-β^+/Δβluc^ mice were s.c. injected with 5x10^6^ pUCΔGDD (ΔGDD) transfected or HCV SGR RNA transfected Huh7.5 cells treated with or without the HCV protease inhibitor telaprevir. At the indicated time points post s.c. injection, luciferin was injected i.v., the luciferase activity measured using the IVIS Spectrum *in vivo* imaging system and the signal quantified. Each symbol represents an individual animal (n = 3–4). (****, p≤ 0.0001, ***, p≤ 0.001; **, P≤0.01; *, P≤0.05; 2-way ANOVA, means + SD; n.s. not significant).

Since Flt3-L derived DC only resemble physiological DC subsets but do display some differences to *in vivo* derived DC, we analyzed whether HCV replication can be sensed and IFN be induced also *in vivo*. To this end, IFN-β^+/Δβluc^ reporter mice, encoding a luciferase gene under the control of the IFN-β promotor [34] were utilized. Mice were subcutaneously (s.c) injected with 5x10^6^ SGR2 transfected Huh7.5 cells (right flank) or 5x10^6^ mock transfected Huh7.5 cells (left flank) and the luciferase activity was measured at different time points post injection. The SGR2 transfected cells induced a significant luciferase signal and therefore IFN-β promotor activity 4 h post injection which decreased moderately at later time points (Fig 6A and 6B). Importantly, the IFN stimulation was dependent on active viral replication, as injection of cells harboring a replication incompetent HCV subgenomic replicon mutant (pUCΔGDD) or treatment with an HCV protease inhibitor abrogated IFN-β promotor activity (Fig 6C). Taken together, adoptive transfer of HCV transfected cells stimulates IFN-β production in replication-dependent manner *in vivo*. Given the subcutaneous route of injection it is possible that not DC but rather Langerhans cells or tissue-resident macrophages are responsible for the observed IFN induction *in vivo*. To address whether murine macrophages are able to sense HCV replication and produce type I IFN we isolated bone-marrow derived macrophages and cocultured them with HCV replicon or virus-transfected hepatoma cells. Coculture did not lead to an IFN-α or IFN-λ production by macrophages and only a moderate IFN-β production (S3 Fig). Most importantly we could show that this IFN-β production was also dependent on TRIF signaling as TRIF knockout rendered the cells unable to respond to HCV coculture (S3 Fig). This demonstrates that HCV replication can be sensed also by other cell types *in vitro* in a TRIF dependent manner, however that Flt3-L derived DC are the main producers of type I IFN.

## Discussion

In this study, we describe basic principles of murine DC activation by HCV replicating cells. In the majority of cases, HCV exposure results in chronic infection with a high risk to develop severe liver diseases like cirrhosis, fibrosis and hepatocellular carcinoma [2]. Despite stimulation of early immune responses within the liver as well as high IFN-stimulated gene expression the virus is able to persist in its host [10, 35]. Dendritic cells act as sentinels of the immune system and constitute a first line of defence against invading pathogens. Although human hepatocytes have been reported to produce type I and type III IFNs in response to HCV infection [36, 37], the virus has evolved evasion mechanisms including the cleavage of the adaptor molecules MAVS and TRIF, thus preventing signal transduction and IFN induction in these cells [38–40]. Therefore, dendritic cells that are not infected by HCV constitute an important defence mechanism to establish an antiviral status. Intrahepatic studies of dendritic cell interactions are technically and practically challenging and the isolation of certain dendritic cell subsets from human peripheral blood mononuclear cells (PBMC) can be difficult. So far only few studies have been conducted using murine derived dendritic cells mainly due to the lack of a suitable immunocompetent murine animal model, which supports HCV replication in *vivo* [41, 42]. Given the fact, that murine Flt3-L derived dendritic cells comprise subsets that are equivalent to physiological DC subsets found in humans, pDC, CD1c^+^ DC (with the murine counterparts CD11b-like DC) and CD141^+^ DC (with the murine equivalents of CD8α-like DC) [23, 43], we used this system to study virus host interactions. Interestingly, only Flt3-L but not GM-CSF derived DC were able to produce type I and type III IFN after co-culture with HCV transfected cells. Similar to what has been described for human pDC and CD141^+^ DC [17, 19, 31], murine IFN-α production was dependent on active viral replication and endocytosis, as co-culture with a replication incompetent virus mutant (ΔGDD) or treatment with acidification inhibitors prevented type I IFN production of DC. Interestingly, cell-free virus as well as purified supernatant harvested from HCV SGR transfected cells was able to stimulate IFN production *in vitro*, indicating that viral signatures are sensed independent of direct cell-to-cell contact and independent of infectious virus production. A recent study suggested that exosomes are released from HCV infected cells that stimulate human pDC to produce IFN-α [44]. Other studies confirmed that SGR RNA can be transferred from one cell to another via exosomes resulting in a productive replication in susceptible cells [45–47]. Indeed, after stimulation of DC with preparations of extracellular vesicles we could observe type I IFN induction. Therefore, our data are consistent with these previous reports and indicate that exosomes and not the virus particle itself deliver signals to murine immune cells and thus trigger IFN production. However, further studies are needed to reveal the detailed mechanism and the features of these exosome-like vesicles. Sensing of HCV-transfected cells was dependent on TRIF and IFNAR signalling and sorting of individual DC subtypes showed that the CD8α-like DC, which are the murine counterpart of human CD141^+^ DC, are the main IFN producers in the murine culture after stimulation with HCV-transfected cells. Interestingly, there are striking parallels between the human and the murine system with the human CD141^+^ DC being able to sense HCV replication in a TRIF dependent manner, to produce IFN-λ and to amplify IFN-α responses [17, 18]. Furthermore, *ex vivo* isolated murine CD8^+^ DC have been described to produce type III IFN in an IPS-1 dependent manner upon stimulation with HCV [42], further supporting our data. However, unlike human pDC [19, 20], isolated murine pDC were not able to sense HCV replication. The reason for this remains elusive and needs to be further studied. One possible explanation is that pDC, in contrast to the other DC subtypes, do not express TLR3 [48]. However, similar to human pDC, recognition of HCV RNA could still be mediated via TLR7 in these cells [19]. Since co-culture with both human and murine HCV replicating liver cells did not stimulate murine pDC it is unlikely that species-specific recognition mechanisms are responsible for the inability of murine pDC to recognize HCV replicating cells.

FACS analyses revealed that in the whole Flt3-L DC culture all cell types were activated after coculture with HCV transfected cells. This is supported by our finding that all cell types (including the pDC cell population), upregulated the activation marker CD69. However, if all cell types contributed to IFN production remains to be determined since the mode of activation may differ between cell subsets. Further experiments are needed to define the contribution of each cell type. Nevertheless, this interplay of cell subsets could resemble the physiological situation within the liver of the infected hosts, where several immune cells are likely to communicate to establish an antiviral state.

Taken together, we have demonstrated the usage of murine BM-derived dendritic cells, which can be easily generated to study virus host interactions and to elucidate signalling pathways which are important for the establishment of an antiviral state during HCV infection. With the lack of an immune competent mouse model to study immune responses to HCV, alternative methods are needed to further elucidate virus-host interactions. We also show that there are conserved functions and similarities between the murine DC and their human counterparts, with respect to HCV sensing, but we could also show differences between both systems. Therefore this model should be useful to further define virus host interactions during HCV infection and to dissect basic principles of intercellular communication between infected cells and DC subpopulations.

## Materials and Methods

### Ethics statement

All mice were bred under specific-pathogen-free conditions at the mouse facility of the Helmholtz Centre for Infection Research, Braunschweig, Germany, or at the Twincore, Centre of Experimental and Clinical Infection Research, Hannover, Germany. All animal experiments were performed in compliance with the German animal protection law (TierSchG BGBI S. 1105; 25.05.1998) and were approved by the responsible state office (Lower Saxony State Office of Consumer Protection and Food Safety) under the permit number 12/1025. The mice were handled in accordance with good animal practice as defined by the Federation for Laboratory Animal Science Associations (FELASA).

### Mice

C57BL/6 mice were purchased from Harlan Winkelmann. IFNAR^-/-^ mice [49], IL28R^-/-^ mice [50], MyD88^-/-^ mice [51], CARDIF^-/-^ mice [52, 53], TRIF^-/-^ mice [54] and IFN-β^+/Δβluc^ reporter mice, encoding a luciferase reporter gene under the control of the IFN-β promoter [34] have been described recently.

### Cell lines and cell culture

MLT-MAVS^−/−^miR-122/mmmmm cells have been described recently [33]. Please note that MAVS and CARDIF are referring to the same gene. Huh-7.5 cells were kindly provided by Charles Rice, Rockefeller University, New York, USA [55]. Huh-7.5 cells and MLT-MAVS^−/−^miR-122/mmmmm cells were cultured in Dulbecco’s modified minimal essential medium (DMEM, Life Technologies) supplemented with 2 mM L-glutamine (Invitrogen), non-essential amino acids (Invitrogen), 100 μg/mL streptomycin (Invitrogen), 100 IU/mL penicillin (Invitrogen), and 10% fetal bovine serum (DMEM complete) at 37°C and 5% CO_2_.

### Generation of bone marrow-derived dendritic cell subsets

Femur and tibia of mice were flushed with DC medium (RPMI medium supplemented with 10% FCS, 10 mM HEPES (Gibco), 1 mM sodium pyruvate (Gibco), 2 mM Glutamax (Gibco), 100 U/mL penicillin (Gibco), 10 mg/mL streptomycin (Gibco), and 0.1 mL β-mercaptoethanol (Sigma)) to isolate bone marrow (BM) cells. Red blood cell lysis was performed using red blood cell lysing buffer (Sigma). Cells were washed, seeded at appropriate densities and incubated for 8 days in the case of dendritic cells and for six days in case of macrophages. For Flt3-L derived DC cultures, cells were seeded at a density of 2x10^6^ cells/mL in DC medium supplemented with 100 ng/mL Flt3-L (R&DSystems) and incubated at 37°C and 5% CO_2_. A medium change was performed at day 4 by replacing two-thirds of the cell culture volume with fresh medium supplemented with Flt3-L. To obtain GM-CSF derived DC cultures, BM cells were seeded at a density of 1x10^6^ cells/mL in DC medium supplemented with 100 ng/mL granulocyte-macrophage colony-stimulating factor (GM-CSF) (R&D Systems), and the medium was changed at days 4 and 6. For M-CSF derived macrophages cultures, cells were seeded at a density of 5x10^5^ cells/mL in 100 μl/mL LCCM (L929 cell conditioned medium) supernatant. A medium change was performed at day 3 by replacing half of the cell culture volume with fresh medium supplemented with LCCM supernatant as described before [56].

### Plasmids and virus production

The plasmids pFK-Jc1 (Jc1) and pFK;i389-LucNS3-3’ (SGR/SGR1) have been described recently [57, 58]. The plasmid pFK-Jc1ΔGDD (ΔGDD), which has an in-frame deletion of 10 amino acids spanning the GDD motif in the NS5B polymerase catalytic domain, has been constructed by cloning the deletion into the pFK-Jc1 plasmid. The cloning strategy is available upon request. The plasmids pFK;i341NeoEI-NS3-NS5B/JFH1 (SGR2) and pUC-pSGR-JFH1/_GDD (pUCΔGDD) have been described elsewhere [59, 60]. For preparation of infectious HCV or subgenomic replicon (SGR) supernatant stocks, Huh7.5 cells were transfected with 10 μg *in vitro* transcribed viral RNA or no RNA (Mock) using electroporation [57]. After 48 h and 72 h incubation, cell-free supernatant (SN) was filtered through 0.45 μm filters, ultrafiltrated through Amicon centrifugal filters (Millipore), and 100x concentrated. For the production of HCV subgenomic replicon (SGR) SN and Mock SN, medium was changed after 4 h to serum-free AEM medium and the supernatant harvested and concentrated as described above. SGR SN contained the following HCV genome equivalents: 1–9 x 10^10^ copies/mL as determined by quantitative real time PCR (qRT-PCR) as described before [61]. For extracellular vesicle isolation, Mock, SGR or pUCΔGDD supernatant was harvested as described above. 1 mL concentrated supernatant was incubated with 250μL ExoQuick-TC solution (SBI) overnight at 4°C and extracellular vesicles recovered according to the manufacturer’s instructions. Vesicle isolation was controlled via western blot according to standard procedures using the following antibodies: anti-Hsp70 (SBI), anti-Annexin II (clone 5, BD), anti-CD81 (JS-81, BD), anti-CD63 (MEM-259, Biolegend) and anti-actin (MAB 1501R, Chemicon) and Horseradish-peroxidase–conjugated secondary anti-rabbit or anti-mouse antibodies (Amersham; SBI). Vesicles contained the following HCV genome equivalents: ΔGDD [2–8 x10^8^ copies/mL], SGR [4–10 x10^9^ copies/mL] as determined by qRT-PCR as described before [61].

### Treatment of cocultures and cell supernatants

For RNAse and DNAse treatments of the coculture, transfected Huh7.5 cells were washed and Flt3-L derived DC were added immediately before 0.5 μg/mL RNAse (Roche) or 1 unit DNAse (Roche) were added and the cells were cocultured for 18 h. For bafilomycin A1 (Bafilo; Sigma) and concanamycin A (ConA; Sigma) treatments, transfected Huh7.5 cells were washed, Flt3-L DC were added and the coculture was treated with 5 nM ConA or 25 nM Bafilo for 18 h.

### Coculture and *in vitro* stimulation of bone marrow-derived dendritic cell subsets

Huh7.5 cells were transfected with HCV RNA constructs, seeded in 96 well plates at a density of 1x10^5^ cells/200 μl, and incubated for 72 h. After 72 h, the cells were washed with 1xPBS before 2x10^5^ cells/200μl DC were added and co-incubated with the cells for 18 h. For stimulation experiments 2x10^5^ cells/200μl DC cultures were seeded in 96 well plates and stimulated with VSV-M2 at a MOI 1, HCV full length virus (Jc1) at a MOI 10 or with 5 μl of concentrated supernatant or isolated extracellular vesicles for 18 h. The cell-free supernatant was harvested and analysed by ELISA and the cells were stained for fluorescent activated cell sorting (FACS) analysis.

### Flow cytometry

For FACS analysis as well as FACS sorting of single-cell suspensions, cells were first incubated with CD16/CD32-specific antibody (2.4G2; BD) for 10 min to block non-specific Fc-receptor interactions. A live death staining was performed using zombie aqua (eBioscience) according to the manufacturer’s instructions. To differentiate between DC subsets, cells were stained for 15 min at 4°C with combinations of antibodies specifically binding to CD11b (M1/70.15; Invitrogen), CD11c (HL3; BD), Siglec-H (eBio440c; eBioscience), CD69 (H1.2F3; BD), Clec9a (42D2; eBioscience), CD172 (SIRPα) (P84; Biolegend) and CD24 (M1/69; Biolegend). The cells were subsequently washed with 1 mL FACS buffer (PBS, 1% FCS) and then re-suspended in FACS buffer supplemented with 3% paraformaldehyde (PFA). Samples were measured using a FACS LSR II, and data were analyzed with FlowJo 7.6.5 software (TreeStar). Dead cells as well as cell duplets were excluded prior to subsequent analysis. DC subsets were sorted using the FACS Aria or FACS XDP and sorting efficiency ranged between 71.9–86.2% for the pDC, 81.4–94.4% for the CD11b-like DC and 44.54–83.2% for the CD8α-like DC.

### Cytokine measurement by ELISA

For the determination of IFN-α, IFN-β and IFN-λ levels in cell-free supernatants, enzyme-linked immunosorbent assay (ELISA) methods were applied (eBioscience, PBL Biomedical Laboratories) following the manufacturer’s instructions. Supernatants were harvested and stored at -20°C until they were tested for IFN. Background lines (dashed lines) depict the lowest standard detected of IFN-α (31.25 pg/mL), IFN-β (15.6 pg/mL) and IFN-λ (62.5 pg/mL).

### Subcutaneous injection of mice and *in vivo* imaging

Huh.7.5 cells were either mock transfected or transfected with the plasmids pFK;i341NeoEI-NS3-NS5B/JFH1 (SGR2) or pUC-pSGR-JFH1/_GDD (pUCΔGDD). For inhibitor treatments, 4 h after transfection a medium change was performed and 1 μM Telaprevir (Selleckchem) was added to the cells. After 72 h, the cells were harvested, washed once with PBS and resuspended as 5x10^7^ cells/mL in PBS. IFN-β^+/Δβluc^ reporter mice were anesthetized with isoflurane, and 5x10^6^ cells were subcutaneous (s.c.) injected into the flanks of the mice. 100 μl luciferin (PerkinElmar) (30 mg/mL in PBS)/20 g mouse weight was intravenously (i.v.) injected at the indicated time points and the mice were immediately analyzed using the IVIS Spectrum CT (PerkinElmer). The acquired images were analyzed and quantified using the Living Image 4.3.1 software (PerkinElmar).

### Statistical analysis

Statistical analyses were done using GraphPad Prism 5. The one-tailed non parametric Mann-Whitney or 2-way ANOVA tests were used for the analysis of significant differences between groups with unmatched pair values. Error bars in graphs indicate standard deviation.

## Supporting Information

S1 Fig Endocytosis inhibitors abrogate HCV dependent type I IFN production by Flt3-L DC. Huh7.5 cells were transfected with HCV subgenomic replicon (SGR) RNA or HCV full length (Jc1) RNA and incubated for 72 h. Cells were washed and murine Flt3-L derived DC were cocultured with mock or HCV RNA transfected hepatoma cells. Co-cultured cells were either left untreated or treated with DMSO, bafilomycin A1 or concanamycin A for 18 h before IFN-α levels were determined by ELISA (n = 3). Dashed line indicates the lowest value of the standard of the respective ELISA assay, n.d. not detected. (*****, p≤ 0.0001, **, p≤ 0.001; **, P≤0.01; *, P≤0.05; 2-way ANOVA, means + SD; n.s. not significant).
(TIF)

S2 Fig Flt3-L derived sorted pDC are not stimulated by HCV replicating murine or human cells. Human Huh7.5 or murine MLT-MAVS−/−miR-122/mmmmm cells were either mock transfected or transfected with two HCV subgenomic RNA constructs (SGR and SGR2). After 72 h, either Flt3-L DC cultures or sorted Flt3-L derived DC were added in a coculture or stimulated with VSV-M2 at a MOI 1 for 18 h and the amount IFN-α measured in the supernatant (n = 3). Human Huh7.5 cells were co-cultured with (A) Flt3-L DC, (B) Flt3-L pDC, (C) Flt3-L CD11b-like DC or (D) Flt3-L CD8α-like DC. Murine MLT-MAVS−/−miR-122/mmmmm cells were co-cultured with (E) Flt3-L DC, (F) Flt3-L pDC, (G) Flt3-L CD11b-like DC, (H) Flt3-L CD8α-like DC. Dashed line indicates the lowest value of the standard of the respective ELISA assay, n.d. not detected. (****, p≤ 0.0001***, p≤ 0.001; **, P≤0.01; *, P≤0.05; Mann-Whitney test, means + SD; n.s. not significant).
(TIF)

S3 Fig Bone marrow derived macrophages are poor producers of interferon after HCV stimulation. Huh7.5 cells were transfected with HCV subgenomic replicon (SGR) RNA or HCV full length (Jc1) RNA and incubated for 72 h. Murine M-CSF derived macrophages were generated from C57BL/6 wildtype mice (A-C) or TRIF knockout mice (D-F) and cocultured with mock or HCV RNA transfected hepatoma cells or stimulated with VSV-M2 at a MOI 1 for 18 h (n = 3). Interferon response was analyzed by ELISA. Analysis of IFN-α (A, D), IFN-β (B, E) and IFN-λ (C, F) in cell-free supernatants of M-CSF derived macrophage cultures. Dashed line indicates the lowest value of the standard of the respective ELISA assay, n.d. not detected. (****, p≤ 0.0001***, p≤ 0.001; **, P≤0.01; *, P≤0.05; Mann-Whitney test, means + SD; n.s. not significant)
(TIF)
