# Supplementary figures and images for: Hepatitis C Virus Stimulates Murine CD8α-Like Dendritic Cells to Produce Type I Interferon in a TRIF-Dependent Manner
Source: PLoS Pathog. 2016 Jul 6;12(7):e1005736. doi: 10.1371/journal.ppat.1005736 (PMC4934921; doi:10.1371/journal.ppat.1005736)

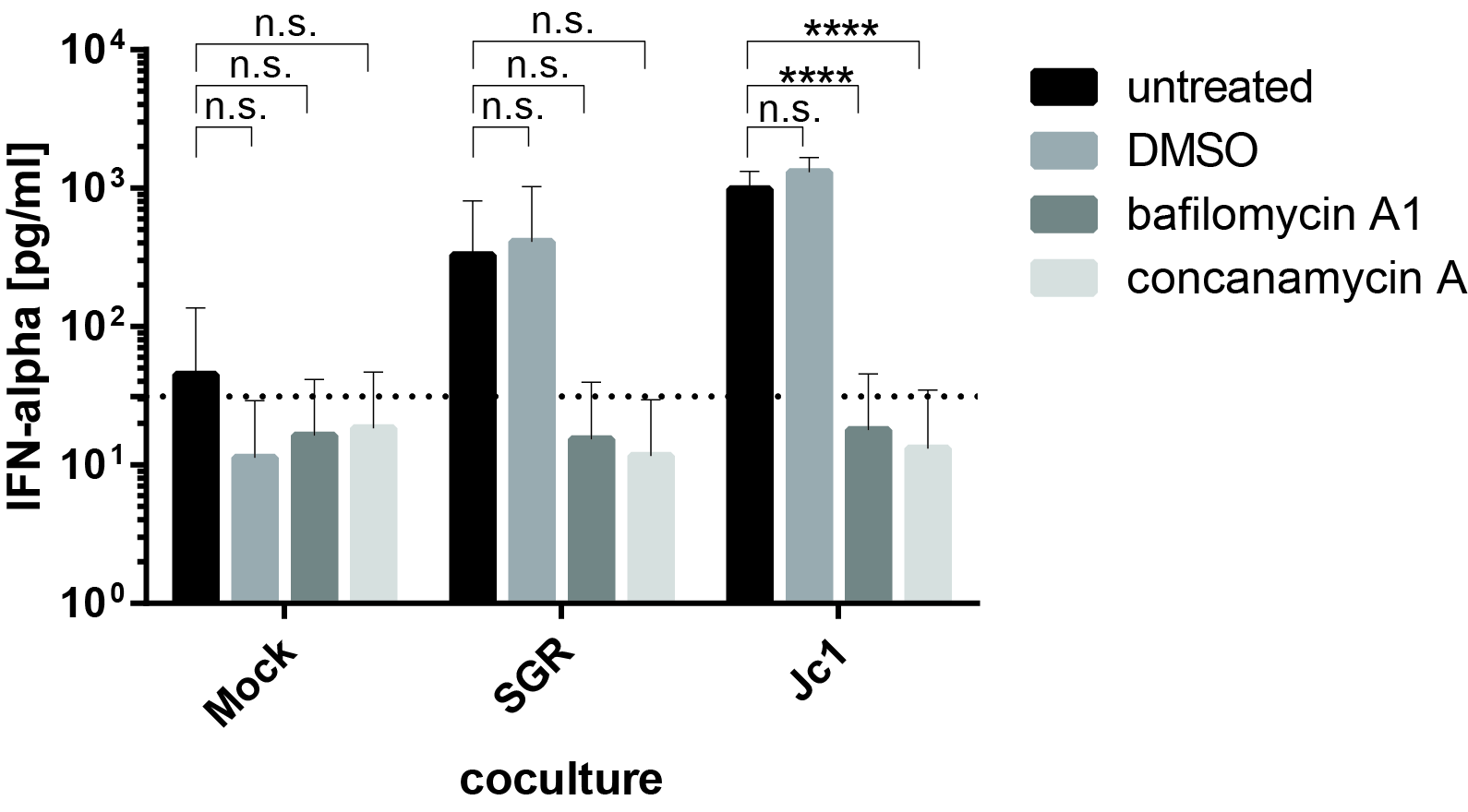

Supplement: S1 Fig — Huh7.5 cells were transfected with HCV subgenomic replicon (SGR) RNA or HCV full length (Jc1) RNA and incubated for 72 h. Cells were washed and murine Flt3-L derived DC were cocultured with mock or HCV RNA transfected hepatoma cells. Co-cultured cells were either left untreated or treated with DMSO, bafilomycin A1 or concanamycin A for 18 h before IFN-α levels were determined by ELISA (n = 3). Dashed line indicates the lowest value of the standard of the respective ELISA assay, n.d. not detected. (*****, p≤ 0.0001, **, p≤ 0.001; **, P≤0.01; *, P≤0.05; 2-way ANOVA, means + SD; n.s. not significant). (TIF) [file ppat.1005736.s001.tif]

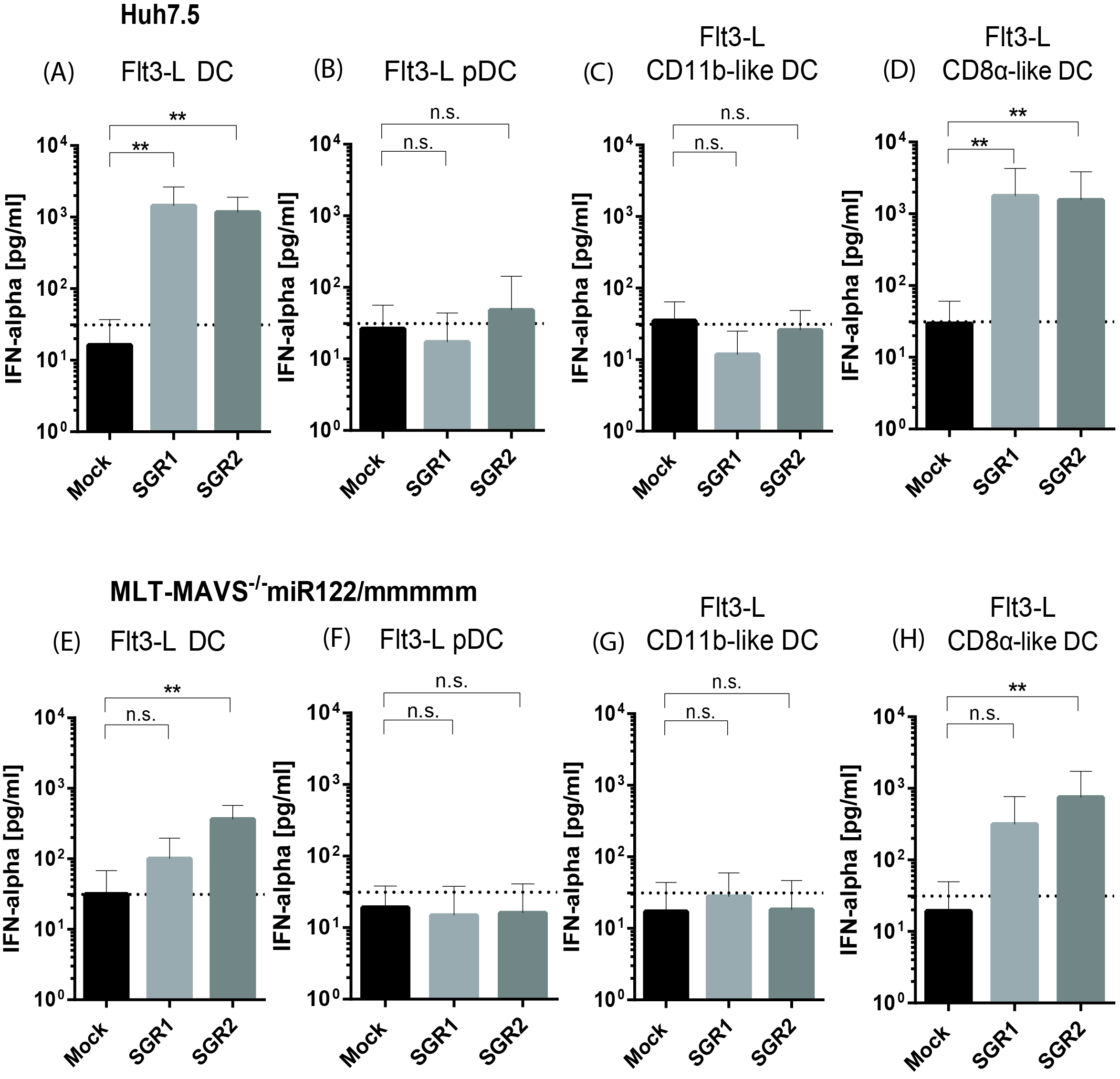

Supplement: S2 Fig — Human Huh7.5 or murine MLT-MAVS−/−miR-122/mmmmm cells were either mock transfected or transfected with two HCV subgenomic RNA constructs (SGR and SGR2). After 72 h, either Flt3-L DC cultures or sorted Flt3-L derived DC were added in a coculture or stimulated with VSV-M2 at a MOI 1 for 18 h and the amount IFN-α measured in the supernatant (n = 3). Human Huh7.5 cells were co-cultured with (A) Flt3-L DC, (B) Flt3-L pDC, (C) Flt3-L CD11b-like DC or (D) Flt3-L CD8α-like DC. Murine MLT-MAVS−/−miR-122/mmmmm cells were co-cultured with (E) Flt3-L DC, (F) Flt3-L pDC, (G) Flt3-L CD11b-like DC, (H) Flt3-L CD8α-like DC. Dashed line indicates the lowest value of the standard of the respective ELISA assay, n.d. not detected. (****, p≤ 0.0001***, p≤ 0.001; **, P≤0.01; *, P≤0.05; Mann-Whitney test, means + SD; n.s. not significant). (TIF) [file ppat.1005736.s002.tif]

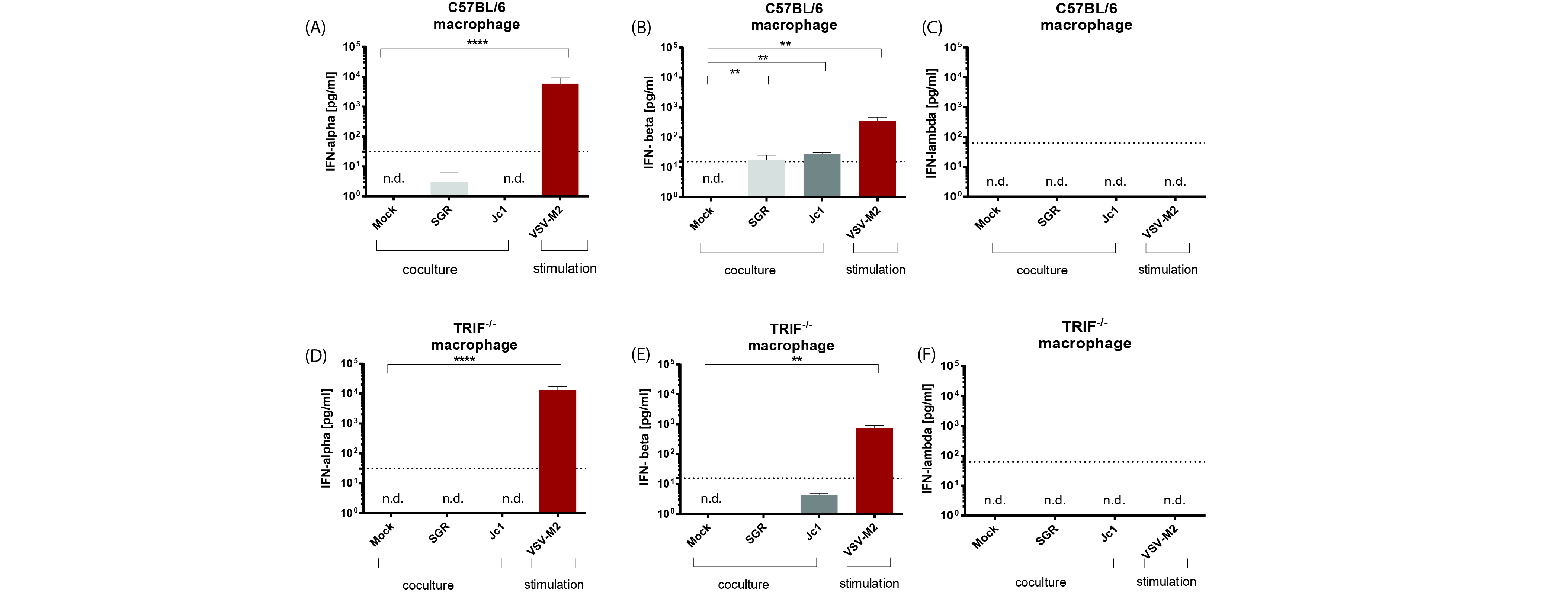

Supplement: S3 Fig — Huh7.5 cells were transfected with HCV subgenomic replicon (SGR) RNA or HCV full length (Jc1) RNA and incubated for 72 h. Murine M-CSF derived macrophages were generated from C57BL/6 wildtype mice (A-C) or TRIF knockout mice (D-F) and cocultured with mock or HCV RNA transfected hepatoma cells or stimulated with VSV-M2 at a MOI 1 for 18 h (n = 3). Interferon response was analyzed by ELISA. Analysis of IFN-α (A, D), IFN-β (B, E) and IFN-λ (C, F) in cell-free supernatants of M-CSF derived macrophage cultures. Dashed line indicates the lowest value of the standard of the respective ELISA assay, n.d. not detected. (****, p≤ 0.0001***, p≤ 0.001; **, P≤0.01; *, P≤0.05; Mann-Whitney test, means + SD; n.s. not significant) (TIF) [file ppat.1005736.s003.tif]
